# Surviving Together by Staying Apart: How Older Adults Experienced the COVID-19 Crisis

**DOI:** 10.1093/geroni/igaa057.1559

**Published:** 2020-12-16

**Authors:** Julie Miller, Lisa D’Ambrosio, Alexa Balmuth, Samantha Brady, Martina Raue, Taylor Patskanick, John Rudnik, Joseph Coughlin

**Affiliations:** 1 MIT AgeLab, Cambridge, Massachusetts, United States; 2 MIT, Cambridge, Massachusetts, United States; 3 Massachusetts Institute of Technology, Cambridge, Massachusetts, United States

## Abstract

The repercussions of novel coronavirus (COVID-19) within the United States and across the globe are vast. Researchers at the MIT AgeLab conducted a national online survey (N=1200) related to COVID-19 in March 2020 with the goal of exploring participants’ COVID-19-related attitudes, hygiene and consumer behaviors, and their use of technology to work, make purchases, stay informed, and stay socially connected. In this presentation, AgeLab researchers will describe findings among a sub-population of study participants deemed to be at highest health risks for the virus: adults ages 60 and over. Findings demonstrate that the largest group of participants stayed informed about COVID-19 primarily through online sources. As a result of the virus, most had made changes to their health and hygiene practices, including implementing social distancing measures, and the majority had changed at least one of their purchasing behaviors, most often buying more products than usual online. Second only to participants’ physical health concerns related to COVID-19 were their financial health concerns, both related to near-term finances and longer-term financial goals. From this study, policy and practice implications emerge across public, private, and non-profit sectors that, taken together, can prepare older adults and their loved ones to survive and thrive during global emergencies.

